# Heterologous expression of newly identified galectin-8 from sea urchin embryos produces recombinant protein with lactose binding specificity and anti-adhesive activity

**DOI:** 10.1038/srep17665

**Published:** 2015-12-07

**Authors:** Kostantinos Karakostis, Caterina Costa, Francesca Zito, Valeria Matranga

**Affiliations:** 1 Consiglio Nazionale delle Ricerche, Istituto di Biomedicina e Immunologia Molecolare “A. Monroy”, Via Ugo La Malfa 153, 90146 Palermo, Italy

## Abstract

Galectin family members specifically bind beta-galactoside derivatives and are involved in different cellular events, including cell communication, signalling, apoptosis, and immune responses. Here, we report a tandem-repeat type galectin from the *Paracentrotus lividus* sea urchin embryo, referred to as *Pl*-GAL-8. The 933nt sequence encodes a protein of 34.73 kDa, containing the conserved HFNPRF and WGxExR motifs in the two highly similar carbohydrate-recognition domains (CRD). The three-dimensional protein structure model of the N-CRD confirms the high evolutionary conservation of carbohydrate binding sites. The temporal gene expression is regulated during development and transcripts localize at the tip of the archenteron at gastrula stage, in a subset of the secondary mesenchyme cells that differentiate into blastocoelar (immune) cells. Functional studies using a recombinant *Pl*-GAL-8 expressed in bacteria demonstrate its hemo-agglutinating activity on human red blood cells through the binding to lactose, as well as its ability in inhibiting the adhesion of human Hep-G2 cells to the substrate. The recent implications in autoimmune diseases and inflammatory disorders make Gal-8 an attractive candidate for therapeutic purposes. Our results offer a solid basis for addressing the use of the new *Pl*-GAL-8 in functional and applicative studies, respectively in the developmental and biomedical fields.

Lectins are proteins that preferentially bind to carbohydrate complexes protruding from glycolipids and glycoproteins^1^. They were shown to bind both to oligosaccharides on cells and to free-floating glycans, at the micromolar to the millimolar range^2^. Due to their binding properties, lectins are involved in cell homeostasis, which is regulated by the controlled transcription of numerous genes. Thus, multiple signalling pathways and enzymatic cascades are influenced by lectins. Therefore, it is not surprising that certain lectins are expressed in a developmentally regulated fashion, both during early embryonic^3^ and postnatal development^4^. A subgroup of lectins is represented by the galectins [previously referred to as ‘S-type lectins’, emphasizing the activity dependence of some of them on thiols (reducing conditions)]^5^. Galectins represent a large ancient family of structurally-related proteins identified in phylogenetically diverse species^6^. Although they have been initially acknowledged as developmentally regulated lectins mainly in vertebrates (amphibians, eel, fish, chick, bovine, rat, mouse and human), galectins have been also identified in sponges and in many protostome and deuterostome invertebrates^7^, (see Supplementary Table S1 online).

Generally physiological functions of invertebrate lectins are not completely understood. Increasing information confirm their involvement in processes like differentiation and development, as well as the elimination of foreign substances through binding to their carbohydrate structures. The Table (S1) lists examples of invertebrates protostome and deuterostome galectins described in the literature, indicating also their location in different cell types and tissues, as well as their putative and proved functions.

Galectins exhibit a carbohydrate binding specificity towards disaccharides containing β-galactosides found in cellular glycoconjugates or glycoproteins^8^. The functional domain(s) of galectins involve one or two carbohydrate recognition domains (CRDs) within a single polypeptide chain. Based on their domain organization, galectin subfamilies are designated as proto-, chimera- and tandem repeat-types^9^. In the tandem repeat type, each one of the two CRDs may have a diverse specificity. Tandem repeat type galectins do not involve diverse functional domains or interaction specificity with non CRDs. This is in contrast to the chimera galectins and the C-type lectins, which often occur together with other domain types in the same peptidic chain^10^,^11^,^12^. Structurally, the galectin’s CRD is a beta-sheet sandwich of about 135 aa, formed by 11 strands; carbohydrates are bound within the groove formed in the concave side by 6 strands^13^. Galectins were initially discovered in tissue extracts and were analysed for their ability to agglutinate erythrocytes; based on the hypothesis that cell surface carbohydrates take part in cell adhesion^14^. They were shown to exhibit inhibitory functions in cell adhesion and to induce apoptosis by binding to integrins^15^. In fact, various functional roles of galectins have been evidenced in cancer^16^, in innate and adaptive immunity^17^ and in development^18^,^19^. Furthermore, galectins appear to be implicated in diverse activities inside and outside the cell, both in invertebrates and vertebrates, where galectins participate in cell adhesion/proliferation, development/morphogenesis, tumor cell metastasis and immune regulation/innate immunity^20^. Although several tandem repeat type members of the galectin superfamily have been described in adult and embryonic tissues of invertebrates^11^ (see Supplementary Table S1 online), no characterization of galectin-8 has been reported for Echinoderms so far. The sea urchin has an extraordinary importance as a model for classical developmental and innovative systems biology studies, being in the lineage leading to the vertebrates and humans^21^. Its genome includes many previously thought to be vertebrate innovations (or known only outside the deuterostomes), providing an evolutionary outgroup for the chordates and yielding insights into the evolution of deuterostomes^21^. The access to the sea urchin genome of the species *Strongylocentrotus purpuratus*^21^ and to ESTs sequences of the species *Paracentrotus lividus*, gave us the opportunity to look for galectin superfamily members. In this study, we report the identification and characterization of *Pl*-GAL-8, a new member of the galectin-8 family isolated from *P. lividus* sea urchin embryo. We describe its: i) cDNA sequence, ii) phylogenetic position, iii) protein domain 3D structure, iv) temporal and spatial mRNA expression profile, v) expression as recombinant fusion protein, vi) carbohydrate-binding specificity and vii) anti-adhesive activity.

## Results

### Identification, cloning and phylogenetic analysis

**Figure 1:**
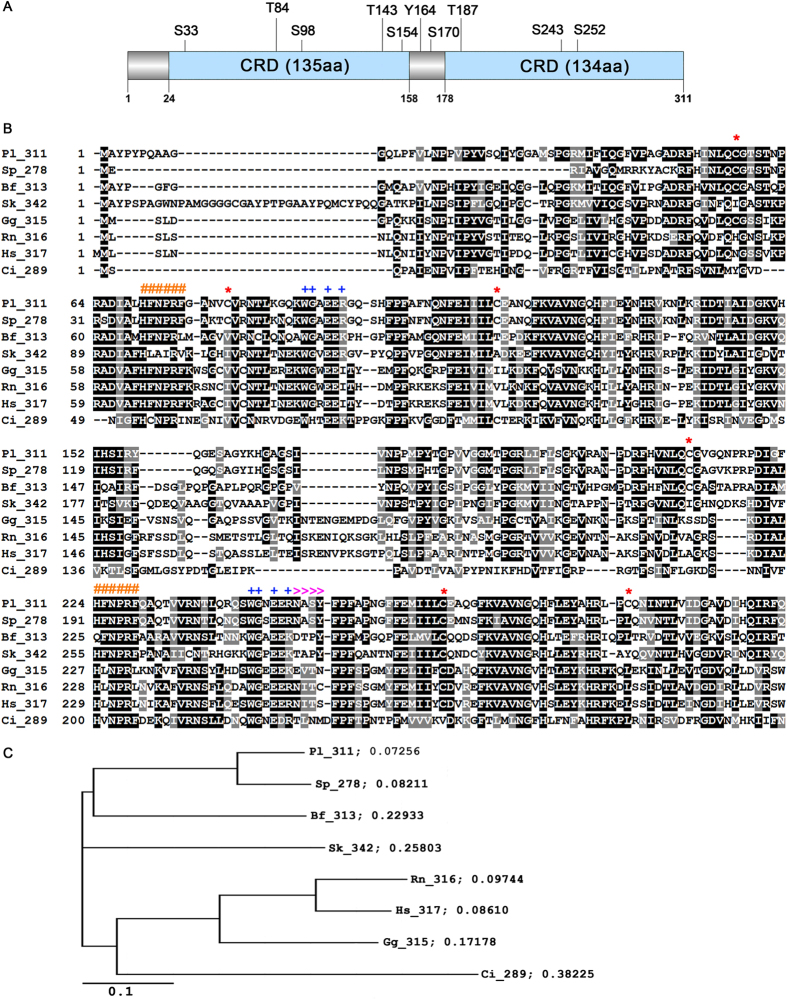
Sequence analysis of *Paracentrotus lividus* Galectin-8. (**A**) Graphic representation of *Pl*-GAL-8 protein structure composed of a 23 aa-long N-terminal region and two tandem repeat CRD domains (24–158 and 178–311), joined by a linker region (159–177). The positions of putative serine, threonine and tyrosine residues are indicated. (**B**) Alignment of the *Pl*-GAL-8 deduced sequence with homologs from: Sp, *Strongylocentrotus purpuratus* (XP_781871); Bf, *Branchiostoma floridae* (XP_002610290.1); Sk, *Saccoglossus kowalevskii* (XP_002731587); Gg, *Gallus gallus* (NP_001010843.1); Rn, *Rattus norvegicus* (AAH72488); Hs, *Homo sapiens* (AAF19370.1); Ci, *Ciona intestinalis* (XP_002126450.1). Numbers after the species acronyms refer to the size in amino acids of the deduced proteins. Identical amino acids are shaded in black, conservative amino acids substitutions are shaded in grey. Hyphenation indicates: (−) gaps inserted for maximizing the match, (*) Cysteine residues; (#) HFNPRF conserved motifs, (+) WGxExR conserved motifs; (≫≫) glycosylation motif. (**C**) Phylogenetic relationships among members of the galectin-8 family. Rooted phylogenetic tree was derived from (**B**). Branch lengths are proportional to the evolutionary distance showing the divergence among different species. The scale bar indicates an evolutionary distance of 0.1 aa substitutions per position in the sequence.

We isolated and cloned the complete *galectin*-8 cDNA from *P. lividus* sea urchin embryos (gastrula stage). The nucleotide sequence has a length of 1309 nt, including a 933 nt-long CDS and a 376 nt-long 3′UTR (Acc. Num. FR716469). The deduced amino acid sequence resulted of 311 residues with a predicted p*I* of 9.39 and a MW of 34.73 kDa. By *in silico* analysis, we identified two carbohydrate-recognition domains (CRD, Fig. 1A); the first spanning from V24 to Q158 (135 aa-long) and the second from Y178 to Q311 (134 aa-long). The two domains are joined by a linker region of 19 aa. In addition, we found ten predicted phosphorylation sites, including six serine residues (S33, S98, S154, S170, S243, S252), three threonine residues (T84, T143, T187) and one tyrosine (Y164) (Fig. 1A). The isolated cDNA showed a high identity (85%) with two sequence*s* previously reported in *S. purpuratus*, namely: the predicted galectin-8-like sequence, of 2038 bp-long mRNA (Acc Num. XM_776778) identified at the NCBI; and the SPU_006306 sequence annotated as *Sp*-Gal/lec3, identified in the Sea Urchin Genome database (http://www.spbase.org/SpBase/search/)^22^. In addition, we found the highest identity with three partial cDNA sequences (98.7%, 90.5%, 92,7%) retrieved from the *P. lividus* EST database (NCBI) embryonic stages libraries (AM551138, AM211139, AM599276), showing sizes of 866, 822, 807 nucleotides, containing 5′UTRs of 170 bp, 90 bp, 199 bp and coding regions of 696 bp, 740 bp, 608 bp. As expected, by comparison of *Pl*-GAL-8 with each of the deduced *P. lividus* amino acid sequences (232 aa, 246 aa, 203 aa) we found the highest values of identity: 99.1%, 92.3% and 98.8%. To be noted that two of the above mentioned sequences (AM211139 and AM599276) contain a linker region of 38 aa, due to presence of a 19 aa additional stretch. Blast search at the NCBI also identified uncharacterised galectin-8 proteins from several phyla including mammals. Apart from the high identity (88% identity and 93% similarity) with the predicted *S. purpuratus* Galectin-8 like (XP_781871), the other identified proteins showed lower percentages of identity, as follows: 54% with *Branchiostoma floridae* (XP_002610290.1), 51% with *Saccoglossus kovalevskii* (XP_002731587.1), 46% with *Gallus gallus* (NP_001010843.1), 45% with *Homo sapiens* (AAF19370.1; AAD45402.1), 43% with *Rattus norvegicus* (NP_446314.2), 42% with *Mus musculus* (NP_061374.1; 40% with *Ostrea edulis* (ADF80416.1)^21^ and 30% with *Ciona intestinalis* (XP_002126450.1).

Figure 1B shows the phylogenetic analysis acquired by ClustalW alignment obtained by comparing *Pl*-GAL-8 with homologous protein sequences of different deuterostomes, from hemichordates to humans. It is noted that with the exception of *G. gallus, R. norvegicus* and *H. sapiens*, all the sequences used in the alignment have only been identified as Galectin-8 homologues, but no characterization of the functional protein domains has been described. As expected, we found the Galectin-8 signature known to be responsible for the unique carbohydrate specificity^23^, in the arginine located at position 64 of the N-terminal domain (R64), which corresponds to R59 of the human Galectin-8. An additional arginine (R204) is found at the same conserved position of the C-terminal domains of *S. Purpuratus*, *B. Floridae* and *S. Kowalevski*. Each of the two *Pl*-GAL-8 domains contains two evolutionarily conserved motifs, HFNPRF and WGxExR, which are present in all galectins described so far. We found six cysteine residues (potential SS intra-, inter- or extra-chain bridges) and one potential glycosylation site (NASY) at position 250–253. Based on the alignment, an NJ phylogenetic tree was generated (Fig. 1C) which demonstrates that among Chordates, Galectins-8 from echinoderms (*P. lividus* and *S. purpuratus)* are phylogenetically closer to Galectins-8 from cephalochordates (Amphioxus).

### Domain homology and modeling

**Figure 2:**
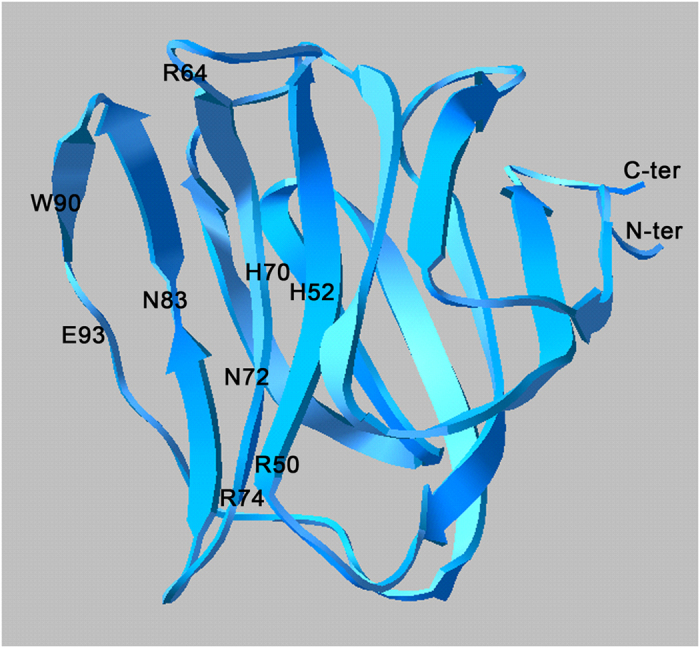
Three dimensional structure model of *Pl*-GAL-8 N-terminal domain. Ribbon model of *Pl*-GAL-8 N-terminal domain obtained by homology modelling using as template the X-Ray structure of the human Gal-8 N-terminal domain (pdb file: 2yv8;). The indicated amino acid residues are carbohydrates-binding sites known^23^ to interact with lactose via hydrogen bonding (R50, H70, N72, R74, N83, W90 and E93) or water-mediated bonds (H52 and R64) and with the galactose ring by *van der Waals* interactions (W90). N-ter, N-terminal; C-ter, C-terminal.

To investigate the homology between the two tandem domains of *Pl*-GAL-8 we aligned their amino acidic sequences using the LALIGN program. As expected, we found a high sequence identity (62.8%) and similarity (83.7%) between domains, suggesting evolutionary-driven gene duplication, typical of most tandem repeated domains. To further characterize the *Pl*-GAL-8 domains, we blasted separately the sequence of each of the two domains at the NCBI and discovered the highest identity (49.6%) and similarity (75.6%) between the N-terminal domains of *Pl*-GAL-8 and human Galectin-8. The comparison of the C-terminal domains showed lower identity (46%) and similarity (63%). It was of interest to study the conservation of the overall 3D structure of the CRD of *Pl*-GAL-8 that is linked to its activity. Up to now, nineteen 3D crystal structures of N- and C- terminal domains of the human Galectin-8 have been deposited at the protein data bank, including structures containing bound carbohydrates or oligosaccharides. Therefore, based on the high primary sequence identity and structural similarity with the PDB structure of the N-terminal domain of human Galectin-8 (pdb: 2yv8), we obtained the 3D model structure of the *Pl*-GAL-8 N-terminal domain (Fig. 2). The model shows a bend-shaped structure, with two opposing sheets, each one containing antiparallel β-strands forming a β-sandwich arrangement. One of the two β-sheets forms a concave region (cleft), containing the conserved carbohydrate-binding sites similarly to the N-terminal CRD domain of human Galectin-8^23^. We found that the N-terminal domain model contains the canonical number of strands, with only differences being in the length of the S5 and S6 strands. In fact, the S5 strand of the *Pl*-GAL-8 model is divided in two smaller strands (S5a and S5b), by the conserved polar amino acid asparagine (N83, corresponding to N79 of the human template), while S5 of the template is undivided. On the contrary, the S6 strand of the *Pl*-GAL-8 model is smaller and identical to the first part (S6a) of the longer S6 strand of the human structure. The latter is subdivided into two smaller S6a and S6b strands by two amino acids, one of which is the conserved glutamic acid (E89) corresponding to E93 found in the *Pl*-Gal-8 model.

By comparison with the human 3D structures (pdb: 2yv8 and 3AP5), we identified nine lactose-binding sites on the *Pl*-GAL-8 domain model. Apart from H52, which in the human Galectin-8 corresponds to Q47, all the rest lactose-binding sites identified in *Pl*-GAL8 are evolutionary conserved. Specifically, the sites: i) R50, H70, N72, R74, N83 and E93, are known to directly interact with lactose via hydrogen bonding^23^, ii) W90, is known to form hydrogen bonds with lactose and participating in *van der Waals* interactions with the galactose ring^23^ and iii) H52 and R64 are known to form a water-mediated hydrogen bond with lactose^23^.

### Temporal and spatial gene expression profiling

**Figure 3:**
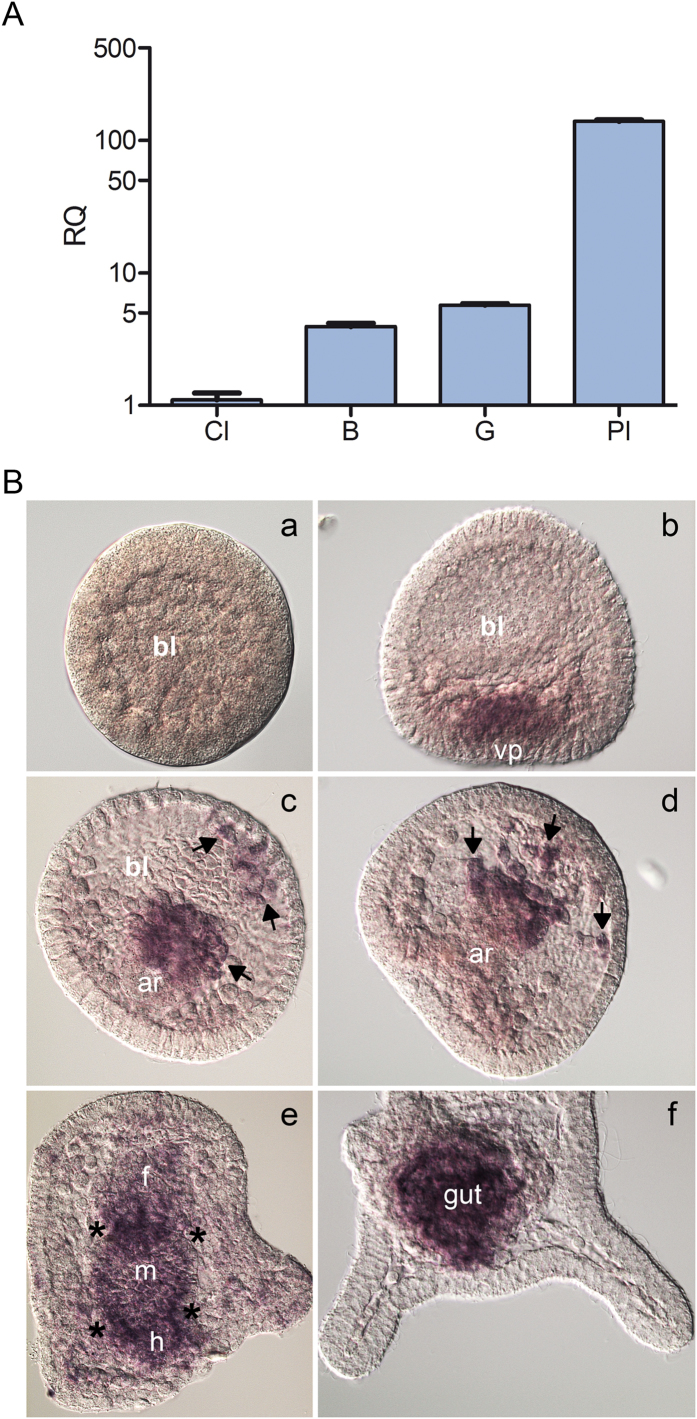
Temporal and spatial expression of *Pl*-*gal*-8 during *P. lividus* embryo development. (**A**) Temporal expression monitored by comparative qPCR analysis, of the *Pl*-Gal-8 transcription levels in sea urchin embryos at different developmental stages: Cl, cleavage; B, blastula; G, gastrula; Pl, pluteus. The *Pl-z12-1* mRNA was used as an internal endogenous reference gene^49^; cDNA from cleavage stage was used as the reference sample and was set as 1 in arbitrary units. The y-axis represents the relative quantity values (RQ or ΔΔCt value, *see Methods*). (**B**) Spatial expression profile studied by whole mount *in situ* hybridization, using a DIG-antisense *Pl*-*gal*-8 RNA probe on embryos fixed at different developmental stages: (**a**), blastula; (**b**), mesenchyme blastula stage; (**c**), (**d**), middle gastrula; (**e**), prism; (**f**), pluteus. Captions: bl, blastocoel cavity; vp, vegetal plate; ar, archenteron; f, foregut; m, midgut; h, hindgut. Arrows in (**c**,**d**) point to the secondary mesenchyme cells; asterisks in (**e**) to the gut sphincters.

The temporal expression profile of *Pl-gal-8* during embryonic development was analyzed by comparative real-time qPCR (ΔCCt qPCR). We found that *Pl-gal-8* mRNA was detectable at the cleavage stage and gradually increased at the blastula and gastrula stages, to reach a peak at the pluteus stage (Fig. 3A). It was particularly important from the developmental point of view to determine if the *Pl-gal-8* transcripts were restricted to specific embryonic territories. To this aim, we analysed the spatial expression of *Pl-gal-8* by WMISH using an antisense RNA probe (containing the complete ORF) on embryos fixed at different developmental stages, from early blastula to pluteus. *Pl-gal-8* transcripts were not noticeable at the blastula stage (Fig. 3B, a) as they were initially detected at the mesenchyme blastula stage, localized at the vegetal plate in a few of the secondary mesenchyme cells (SMCs) and at the presumptive endodermal cells (Fig. 3B, b). At gastrula, transcripts were localized at the tip of the archenteron, which invaginates from the vegetal plate and in some blastocoelar cells at the archenteron tip originated from the detachment of SMCs (Fig. 3B, c–d, see arrows). At the prism stage *Pl-gal-8* mRNA was detected in the three differentiated parts of the gut (foregut, midgut and hindgut), with a higher signal at the level of the two sphincters, namely the constrictions separating the foregut from midgut, and the midgut from hindgut (Fig. 3B, e; see asterisks). No other territories appeared labelled at the pluteus stage, which was the latest stage investigated, where the *Pl-gal-8* mRNA was highly restricted to the gut (Fig. 3B, f).

### Expression and purification of the recombinant protein

**Figure 4:**
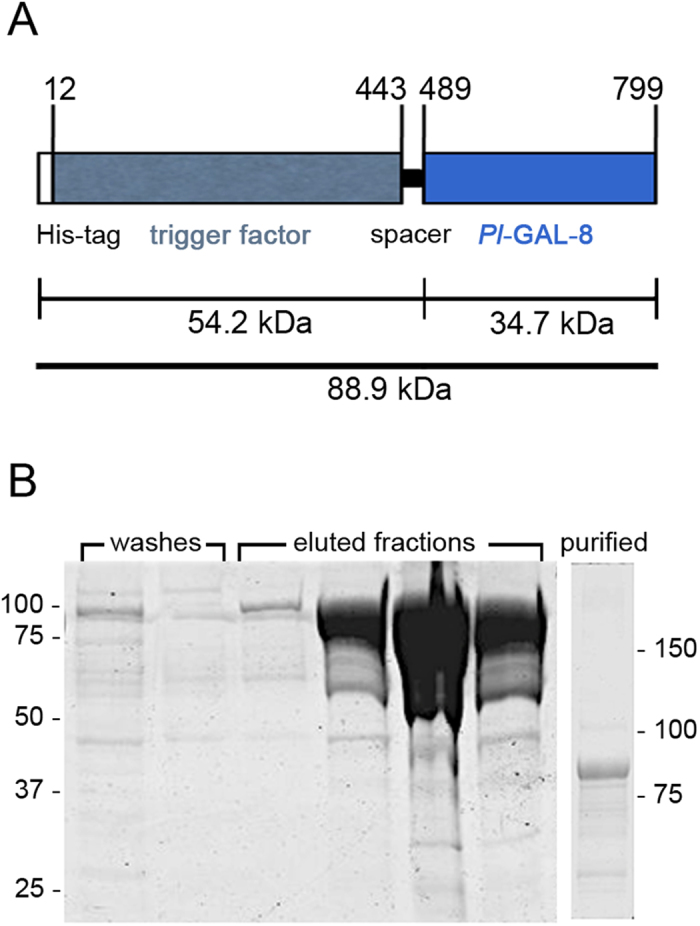
Purification of recombinant *Pl*-GAL-8 protein. (**A**) Schematic representation of the recombinant *Pl*-GAL-8 fusion protein of 799 aa, including a N-terminal His-tag, the trigger factor chaperon protein, a 45 aa spacer and the *Pl*-GAL-8 protein. Numbers on top of the bar indicate amino acids of the construct. The calculated sizes of the protein segments expressed in kDa are indicated underneath. (**B**) SDS-PAGE (7.5%) of washes and eluted fractions from the Ni-nitrilotriacetate affinity chromatography of extracts containing the fusion protein from *E. coli* transformed by the recombinant p*cold-TF-Pl-gal-8* plasmid. Molecular weight markers are indicated on the left. In the separated lane on the right, the purified fusion protein obtained; molecular weight markers on the right.

To produce *Pl*-GAL-8 recombinant protein we constructed a fusion protein by inserting the full-length CDS of *Pl-gal-8* into the p*Cold* vector containing the CDS of the chaperone trigger factor (TF), generally used for the production of soluble and functional/active proteins. It is also known that the TF assists in producing the appropriate folding of recombinant proteins^24^, especially at the most proximal part of the inserted sequence^25^. The construct also contained at the N-terminal an 11 aa-long 6× His-tag, which is needed for protein detection and purification (Fig. 4A). We analysed the bacterial extracts containing the fusion protein by SDS-PAGE, as shown in Fig. 4B. A prominent band was detected in the eluted fractions of the affinity chromatography, indicating an apparent protein size consistent with a theoretical molecular weight of 88.9 kDa, calculated for the fusion protein. The purified recombinant protein obtained from the pooled fractions was then purified by dialysis to reduce salt concentration and remove low molecular weight compounds such as imidazole, which could eventually interfere with the protein folding and activity (Fig. 4B, lane on the right).

### Carbohydrate binding activity and specificity

**Figure 5:**
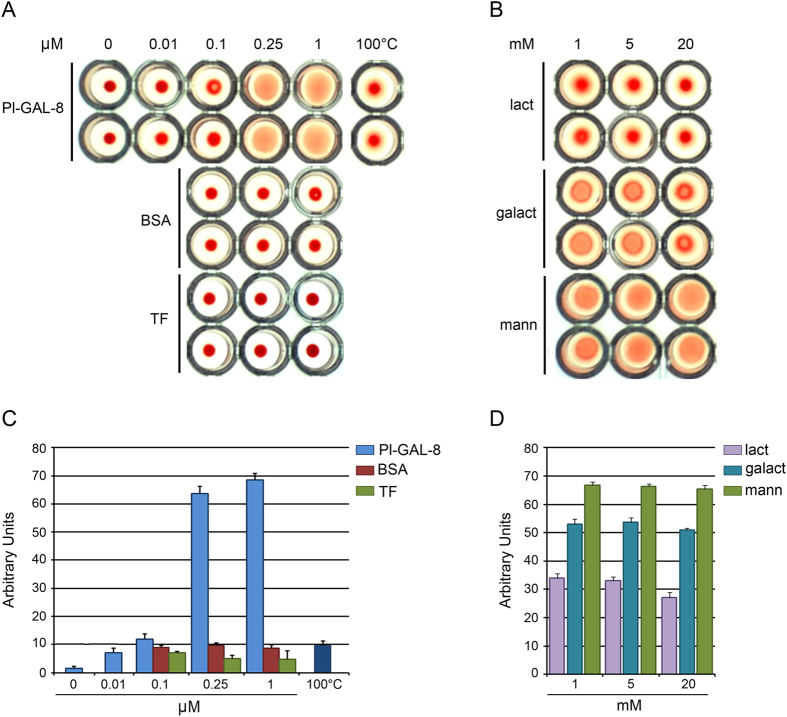
Carbohydrates-binding activity of recombinant *Pl*-GAL-8. Hemoagglutination assays on human red blood cells run in duplicates in U-shaped wells. (**A**) Inhibition of hemo-agglutination was tested by the addition of increasing micromolar (µM) concentrations of: recombinant *Pl*-GAL-8; BSA, serum bovine albumin; TF, trigger factor; 100 °C, 1 μM of recombinant *Pl*-GAL-8 denatured at 100 °C for 5 min before testing (negative control). (**B**) Interaction of mono- or di-sacharide sugars with recombinant *Pl*-GAL-8 (0.25 µM) at the indicated millimolar concentrations (mM). Captions: lact, lactose; galact, galactose; mann, mannose. Wells showing spread RBC exhibit agglutination activity, wells with small red dots lack agglutination ability. Results shown in (**A**,**B**) describe a representative experiment; (**C**,**D**) report the mean of 3 replicates from 3 different experiments, +/− SD, expressed in arbitrary units.

To identify the biological activity of *Pl*-GAL-8, we tested the ability of the recombinant protein to induce agglutination of RBCs in a conventional assay^26^ (Fig. 5). Following 40 min incubation at room temperature, the agglutination activity was determined: wells that contain spread RBC exhibit agglutination activity, while wells that contain small red dots lack agglutination ability. Using serial dilutions, we found that the minimum concentration able to induce agglutination was 0.25 μM (Fig. 5A,C). Loss of *Pl*-GAL-8 activity was evident after the inactivation of the recombinant protein at 100 °C for 15 min (Fig. 5A,C). BSA, also used as a negative control, did not induce RBC agglutination even at the highest dose tested (1 μM, Fig. 5A,C). The purified trigger factor (TF) recombinant protein, obtained from the expression of the p*Cold* vector alone, was also tested and shown to be inactive, thus demonstrating that it does not contribute to the activity of the *Pl*-GAL-8 recombinant protein (Fig. 5A,C). As tandem repeat galectins do not show the tendency to form dimers, trimers, etc.^27^, we infer that the observed carbohydrate binding activity is due to the single recombinant *Pl*-GAL-8 polypeptide. The high similarity of *Pl*-GAL-8 with other members of the family suggests a sugar-binding specificity. To investigate the specificity, we studied the ability of increasing concentrations of different mono- and di- saccharide sugars to interfere with the hemoagglutinating activity of 0.25 μM recombinant *Pl*-GAL-8 (Fig. 5B,D). The agglutination of RBC cells induced by *Pl*-GAL-8, was efficiently inhibited by lactose at all doses tested (from 1 to 20 mM) and, to a certain extent, by galactose at 20  mM. No inhibition of *Pl*-GAL-8 activity was observed with mannose or other sugars tested (glucose, sucrose, maltose; not shown).

### Anti-adhesive activity of the recombinant *Pl*-GAL-8

**Figure 6:**
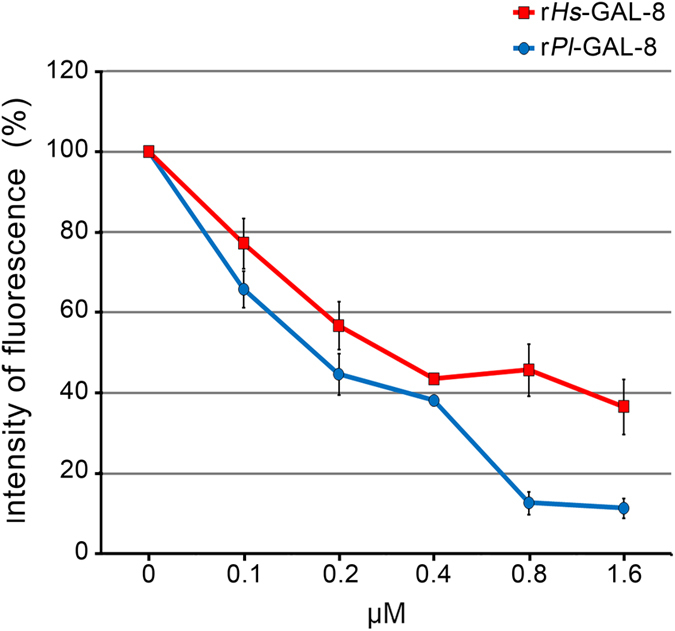
Inhibition of HepG2 cells adhesion to culture plates by soluble *Pl*-GAL-8. Cells were incubated at 37 °C for 3 h in the presence of increasing micromolar concentrations of soluble recombinant human Galectin-8 (r*Hs*-GAL-8, red line) or recombinant *P. lividus*-GAL-8 (r*Pl*-GAL-8, blue line). The percentage of cells that remained bound to the wells bottom was determined by measuring the intensity of auto fluorescence developed after several PBS washings and 0.5% glutaraldehyde fixation. Measurements of fluorescence intensities obtained in arbitrary units, are expressed as percentage of adherent cells. Values are presented as the mean of three replicates from a representative experiment and ± SD, standard deviation.

To further characterize the functional activity of *Pl*-GAL-8, we used the recombinant protein to test its ability to modulate cell adhesion, as described for other Galectins-8^15^. Generally, when immobilized onto matrices (or on well surfaces), galectin-8 supports the adhesion of cells to the substrate, promoting their spreading and migration, triggering integrin-mediated signaling cascades which involve phosphorylation events of intracellular mediators^19^,^28^. On the contrary, when it is present in excess as a soluble ligand, galectin-8 negatively regulates cell adhesion, inhibiting cell-substrate adhesion, by binding both cell surface integrins and other soluble ECM proteins, such as fibronectin^19^,^28^. We tested this second biological activity of the recombinant protein using a human hepato-carcinoma cell line (HepG2) by measuring their adhesion on culture multiwell plates, in the presence of increasing concentrations of soluble recombinant *Pl*-GAL-8. As shown in Fig. 6, recombinant *Pl*-GAL-8 effectively inhibits the adhesion of cells in a dose-dependent manner, as assayed 3 h after plating. About 50% of inhibition was observed at the concentration of 0.2 μM, while only 10% of cells adhered to the wells with the highest concentration used (1.6 μM). As no interfering activity of the trigger factor was detected in the hemoagglutination assay, we decided not to include such a negative control. On the contrary, we used the human recombinant Galectin-8 as a positive control, and found a similar adhesion inhibitory effect (Fig. 6).

## Discussion

In the present study, we report the identification of a new member of the galectin superfamily found in the sea urchin embryo. We describe the identification of the CDS, and the tempo-spatial mRNA expression during development. Additionally, we present the phylogenetic analysis of the protein, characterize its structure and study functional aspects using a recombinant protein we produced. Based on *in silico* analysis and sequence comparison with known homologs from distant phyla, the protein has been identified as a novel member of the galectin-8 family and named *Paracentrotus lividus* galectin-8 (*Pl*-GAL-8).

Members of the galectin family are widely distributed in nature, in both embryonic and adult organisms, implying their importance in carrying out intra- and extracellular functions mediated by glycoconjugate recognition. The molecular characterization of new galectin-8 members was particularly awaited as they are recognized as important actors of many vital functions such as in innate and adaptive immunity and in physiological cell cycle homeostasis^17^. The presence of galectin proteins in adults (coelomic fluid) of the sea urchin *Strongylocentrotus purpuratus* has been reported by proteomic analysis^29^, although no further characterization was described. This is the first report describing the discovery of a galectin superfamily member in Echinoderms, which is added to the short list of invertebrate galectins, (see Supplementary Table S1 online). By identifying a new galectin-8 family member from the sea urchin embryo, a well established model for classical developmental studies, this work contributes information on the galectin-8 family phylogenesis. On the basis of *in silico* cDNA analysis, we hypothesize the presence of other *Pl*-GAL-8 isoforms, probably generated by alternative splicing, as it occurs in mouse and human^29^. The occurrence of different-sized linker regions present in homologous sequences identified in the sea urchin EST database is in favor of this hypothesis. However, further studies will be necessary to demonstrate the existence of more than one sea urchin GAL-8 protein isoform.

In this study we found that *Pl*-GAL-8 does not contain a typical signal peptide, in analogy with other previously identified members of the family known to be targeted not only to cytosolic and nuclear sites, but also to the extracellular environment^28^ where they play key-roles as soluble mediators of various cell functions. Indeed, earlier studies demonstrated that Galectins might be secreted by a non-classical secretory pathway^6^ and later reports on Galectin-1 and Galectin-5 demonstrated that the so-called alternative pathway of secretion implies the involvement in the exosomal sorting pathway^30^.

The lack of a signal peptide in all the Galectins analyzed so far is an additional demonstration of the strong conservation across vertebrate and invertebrate species. In general, the occurrence and the relative positions of cysteine residues are recognized as important factors in both protein structure and function. This is because of their ability to form intra and inter-molecular disulfide bridges influencing the protein folding, thus affecting protein’s functionality. The Galectin-8 proteins from different species compared in this study (Fig. 1B), differ in the total number of cysteine residues. In an evolutionary context, it is worth mentioning that the C268 found in *Pl*-GAL-8 is the most conserved cysteine, as it is found in all the aligned sequences, (with the only exception of galectin from *C. intestinalis*). Generally, the activity of proteins can be modulated by post-translational modifications, such as glycosylation and phosphorylation. In the *Pl*-GAL-8 sequence we found one potential glycosylation motif (NASY) and 10 predicted phosphorylation sites (see Fig. 1B). However, other studies reported that native Galectin-8 is not heavily glycosylated, neither extensively phosphorylated^28^. In agreement, our studies demonstrate that the protein’s biological activity does not depend on post-translational modifications that are obviously not present in the recombinant protein made in bacteria.

The two HFNPRF and WGxExR motifs present in the CRD domains are involved in sugar binding and intra-molecular linking mediated by hydrogen bonds or weak *van der Waals* forces^8^,^10^. Interestingly, we found that the two *Pl*-GAL-8 CRDs (N- and C-terminal) contain identical HFNPRF and WGxExR motifs, unlike human Galectin-8, where CRDs contain sequence differences^28^ that could determine differences in carbohydrate-binding specificity^31^. As a result, our findings suggest that in sea urchins, the N- and C- terminal domains of *Pl*-GAL-8 have the same carbohydrate-binding specificity. This was confirmed experimentally by the heamoagglutination assay.

The N- and C-terminal CRDs of *Pl*-GAL-8 show high values of identity (60%), in contrast to tandem repeat type galectins from other organisms, including human (38%), generally ranging from 20% to 40%,^28^,^32^. Thus, the high CRDs identity suggests that *Pl*-GAL-8 is an ancestral form of the Galectin-8 protein. This is in agreement with the hypothesis proposing that all CRDs of tandem repeat galectins found in chordates evolved by a gene tandem duplication event of an ancestral mono-CRD galectin followed by divergent evolution^33^. In accord, we found that the entire *Pl*-GAL-8 protein showed very high identity values only with *S. purpuratus* (88%) and low-medium identity values (30%–54%) with other organisms. This evidence, together with the high similarity between the two CRDs supports the hypothesis that *Pl*-*gal-8* is the nearest gene to the postulated duplication event.

As for other proteins, the 3D structure of Galectins provided with information on the highly evolutionarily conserved interaction sites. Many crystal structures have been reported for N-terminal and C-terminal domains of the human Galectin-8, alone or in association with sugars or other binding molecules^31^. In general, Galectins are characterized by 11 beta-strands forming two antiparallel sheets. In particular, the sheet forming the concave side of the structure contains six (S1–S6) antiparallel β-strands while the other sheet forming the convex side contains five (F1–F5) antiparallel β-strands^23^,^34^. The finding that the S5 strand of the *Pl*-GAL-8 model is divided into two smaller S5a and S5b strands implies that this feature is irrelevant with respect to the biological function of the molecule, as confirmed experimentally by the hemoagglutination assay (see Fig. 5).

Despite the above mentioned differences, the generated *Pl*-GAL-8 model is highly similar to the structure of the template (human), showing that in both proteins, the conserved sugar-binding cleft exposes the same nine functional residues, with the exception of the histidine (H52) found on the S3 strand, corresponding to the glutamine (Q47) of the human template. A similar difference is found when comparing the sequences of the C-terminal domains, where histidine (H206) in *Pl*-GAL-8 is replaced by asparagine (N215) in human Galectin-8^23^. However, the presence of the conserved histidines (H52 and H206) in *P. lividus*, is indicative of the great homology between the N-terminal and C-terminal domains, confirming their high conservation and calling for their identical carbohydrate-binding specificity. This suggestion is strengthened by the presence of the arginines (R64 and R204) in conserved positions in both N- and C- domains.

In this study, we established a protocol for the expression of a fusion recombinant protein using the cDNA from a marine invertebrate species to address the biological function(s) of *Pl*-GAL-8. The recombinant fusion protein, tested *in vitro* by a hemagglutination assay on human red blood cells and an adhesion assay on human hepatoma cells (HepG2), demonstrated a sugar-binding specificity and its involvement in mediating cell adhesion. This is in agreement with the functional characterization of the Galectin-8 family members described so far, known to act as physiological modulators of cell adhesion^15^,^19^. As expected for a Galectin-8 family member^15^, only lactose (low concentrations) and galactose (higher concentrations), bind to *Pl*-GAL-8 and favor RBC hemagglutination, therefore preventing the inhibitory activity of *Pl*-GAL-8. The low effective inhibitory concentration of lactose (1 mM) suggests a high specificity of the *Pl*-GAL-8-lactose interaction, probably reflecting the bivalency of the protein.

Galectin-8 has a dual effect on the adhesive performance of cells, i.e. depending on the extracellular context it can either promote or inhibit adhesion^15^,^19^. We demonstrated that the recombinant *Pl*-GAL-8 is involved in cell-matrix interactions as it inhibited the binding of HepG2 cells to the substrate in a dose-dependent manner. This finding is in agreement with reports on the mammalian galectin-8^15^,^19^,^28^,^32^. The inhibition was more effective than the one obtained by the highest concentration of human recombinant Galectin-8 used as a control. This result suggests a potential use of the recombinant *Pl*-GAL-8 protein in industrial applications. As for the function of the protein in the sea urchin embryo, the availability of the recombinant *Pl*-GAL-8 encourages future studies, involving for example the production and use of specific antibodies for embryonic perturbation and cell adhesion assays. Although at present we have no direct evidence for *Pl*-GAL-8 adhesion activity in the embryo, a recent study on the proteome of *P. lividus* tube feet (the adhesive organs of the adults) has proposed a role of Galectin-8 in mediating temporary cell-substrate adhesion^35^.

The temporal and spatial expression patterns of *Pl*-GAL-8 during sea urchin development have been described here for the first time. By qPCR, we found that *Pl*-*gal-8* transcripts showed a developmental upregulation at gastrula and pluteus stages. This finding is in accordance with the important role attributed to galectins in controlling morphogenetic events during embryogenesis^36^ and particularly with the role assigned to carbohydrates and carbohydrate-bearing molecules in supporting cell-cell interactions during sea urchin gastrulation^37^. For example, the binding of secondary mesenchyme cells to the blastocoel roof is prevented by D-mannose like residues, resulting in the inhibition of gastrulation^37^. This finding matches with the spatial localization of *Pl*-*gal-8* transcripts at the tip of the archenteron found by WMISH in this study. The staining observed at the tip of the invaginating archenteron is associated to two major presumptive cells types, i.e. blastocoelar cells and gut cells, originating at the vegetal plate of the embryo from mesoderm and endoderm germ layers^38^. Blastocoelar cells leave the tip of the archenteron and remain localized within the blastocoel cavity where they spread and form a network of cells connected by cytoplasmic filopodia. Subsequently, they surround the gut and also localize along the skeletal rods within the arms of the pluteus larva^39^. We have no evidence of *Pl*-*gal-8* transcripts along the skeleton of the embryo, at any stage of the development. To date, very few studies have explored the role of blastocoelar cells. Their involvement in phagocytosis and the expression of innate immunity genes has been established, suggesting a function in embryonic immunity^21^,^40^,^41^,^42^. In analogy with what was recently reported in the adult sea urchin, where immune response genes and proteins have been found in cells dispersed within all major organs, including the gut^43^, we suggest that *Pl*-*gal-8*-expressing cells of mesodermal origin (blastocoelar cells) are probably interspersed in the larval gut, later in development (in pluteus).

Galectin-8 has also pro-apoptotic activity, often involving the activation of the caspase-3 and ERK pathways^44^. The expression of *Pl*-Gal-8 in the gut at the pluteus stage matches with the physiological high apoptotic activity observed in previous studies in gut cells^45^.

The finding that *Pl*-GAL-8 transcripts are found more concentrated around the two sphincters of the gut, probably associated to myoblasts forming the intestinal musculature, is reminiscent of a similar pattern observed by scanning electron microscopy and anti-actin immunofluorescence in *S. purpuratus* embryos^46^. The discovery of *Pl*-GAL-8 in the gut might serve in future work to unravel the complex morphogenesis occurring in the sea urchin embryo developing gut, well-illustrated in a recent article reviewing ultrastructural patterns and dynamics of gene expression^47^.

Despite that the sea urchin genome is sequenced, the many studies at the gene level, and the complex gene regulatory networks investigated so far with great accuracy in the embryo^21^, the function of many proteins expressed during development is not very well known and understood. The success in the production of biologically active recombinant proteins will allow addressing key questions related to *Pl*-GAL-8 involvement in cellular communication and signaling. The finding that the recombinant fusion protein is more effective than the commercially available one of human origin is also interesting for potential pharmacological and therapeutic applications. The production and exploitation of galectins in analogy to proteins currently isolated from marine animals^11^, is a fast growing field in life sciences, due to the potential physiological, biological and pharmacological uses. In conclusion, this work is a contribution to the evolutionary knowledge of the galectins expressed in marine invertebrate embryos and a hint to use the recombinant *Pl*-GAL-8 for industrial purposes in the biomedical field.

## Methods

### Embryo culture, total RNA extraction and RT-PCR

Adult sea urchins (*P. lividus*) were collected from the North-Western coast of Sicily (Mediterranean Sea) and kept in aquaria with circulating seawater obtained from the collection site. Spawning was induced by intra-coelomic injection of 0.5 M KCl. After fertilization, embryos (4000/ml) were cultured in glass beakers in Millipore-filtered sea water containing antibiotics (50 mg/L streptomycin sulfate and 30 mg/L penicillin) with gentle stirring at 18 °C. Unfertilized eggs or embryos at different developmental stages were collected by low-speed centrifugation, instantly frozen in liquid nitrogen and stored at −80 °C for subsequent RT-PCR experiments or fixed in 4% paraformaldehyde in seawater and stored at −20 °C for whole mount *in situ* hybridization (WMISH) experiments as previously reported^48^,^49^. Total RNA from approximately 4000 embryos was isolated using the Gene Elute Mammalian total RNA kit (Sigma). Residual DNA was digested by DNase I (Ambion) according to the manufacturer’s instructions. Target cDNA was directly amplified from the total RNA in a one-step reaction using the SuperScript One-Step RT-PCR kit (Invitrogen), following the manufacturer’s instructions.

### Cloning of *Pl-galectin-8*

We used a sequence predicted to code for a Galectin protein in the sea urchin *Strongylocentrotus purpuratus* database (accession number XP_781871.1) for BLAST screening against *P. lividus* EST databases available at NCBI and MPIMG (http://goblet.molgen.mpg.de/cgi-bin/webapps/paracentrotus.cgi). Three overlapping *P. lividus* EST clones were identified and used to design specific primers (MWG, Heidelberg, Germany). Purified total RNA extracted from the gastrula stage was used to amplify by One Step RT-PCR (Invitrogen), a partial sequence of the 5′ CDS region. We used a 3′RACE kit (Invitrogen) to identify the 3′-terminal end of the sequence. The amplification products were cloned in the p*GEM-T-Easy* Vector (Promega) and sequenced (MWG, Heidelberg, Germany). The full-length 1309nt sequence was deposited to the EMBL databank, Acc Num: FR716469. New primers were designed to obtain by RT-PCR the amplification of the complete CDS (5′-ATGGCATACCCATACCCACAAGC-3′ as forward and 5′-GCACCCGAAAAATCATCCCTAC-3′ as reverse), which was cloned in the p*GEM-T-Easy* vector (Promega) and re-sequenced for validation. The obtained recombinant plasmid was referred to as p*GEM-T-Easy-Pl-galectin-8*.

### *In silico* characterisation, phylogenetic analysis and structural modelling

The deduced amino acidic sequence was analysed *in silico* using different tools found at the servers http://web.expasy.org, http://www.cbs.dtu.dk/services/, as described previously^50^. Phylogenetic analysis of the amino acidic sequence was performed by ClustalW alignment and by the Neighbor-Joining phylogenetic tree as described by^50^. To spot the best local alignment among sequences we used BLAST analysis and the LALIGN program (http://www.ch.embnet.org/software/LALIGN_form.html).

The Modeller software (http://salilab.org/modeller/) was used to obtain the 3D structure model of the N-terminal domain of *Pl*-GAL-8, based on the 2yv8 crystallographic structure of the human Galectin-8 N-terminal domain, retrieved from the protein data bank (PDB) (http://www.rcsb.org/pdb). The model is presented using the PDB Viewer program, as previously described^50^.

### Comparative Real Time qPCR

Gene expression was measured by q-PCR with the Comparative Threshold Cycle Method and SYBR Green chemistry using the Applied Biosystems Step One Plus real time PCR cycler, following the manufacturer’s instructions. cDNAs were synthesised using as templates total RNAs extracted from different stages (cleavage, blastula, gastrula, pluteus) and random hexamers, as described in the MultiScribe Reverse Transcriptase protocol (Applied Biosystems). *Pl-gal-8* primers were designed using the Primer Express software (v2.0.0; Applied Biosystems, Foster City, CA, USA) to amplify a fragment of 82 bp. The primers were: 5′-AAACATGGAGCTGGCAGCAT-3′ as forward and 5′-TGAGTCTCCCTGGAGTCATTCC-3′ as reverse. The ΔΔCt method was used: the mean and standard deviation was calculated for each sample and the values were normalized with their respective endogenous control results to obtain the ΔCt values. Then, the normalized ΔCt values were compared to the reference sample (cleavage stage) set to 1 in arbitrary units, to obtain the relative quantity values (RQ or ΔΔCt value y-axis). The *Pl-z12-1* mRNA was used as an internal endogenous reference gene^49^.

### Whole-mount *in situ* hybridization

The *pGEM-T-Easy-Pl-galectin-8* plasmid was used as template for the synthesis of Digoxygenin (DIG) labelled antisense and sense RNA probes. Whole-mount *in situ* hybridization was performed in a 96-well plate (Greiner Labortechnik, Longwood, FL, USA), using 30–40 embryos per well as previously described^49^. The hybridization reactions were carried out at 62 °C, for at least 20 h. After hybridization, specimens were extensively washed and incubated with an anti-DIG alkaline phosphatase-conjugated antibody (Roche). Immuno-detection was carried out at room temperature with BCIP/NBT chromogenic substrates. Stained embryos were mounted on glass slides and observed under a Zeiss Axioscop 2 plus microscope. Images were captured by a digital camera. Control hybridization reactions with the sense probe did not show any specific signal.

### Production of recombinant *Pl*-GAL-8 protein in *E. coli*

The complete CDS was amplified from the *pGEM-T-Easy-Pl-galectin-8* recombinant plasmid by PCR using the primers: 5′-GGACATATGGCATACCCATACC-3′ as forward and 5′-GCTCTAGATTACTGGAAGCGAATC-3′ as reverse, containing the *Nde*I and *Xba*I restriction sites. After digestion, the amplified product was cloned in the p*COLD-TF* (TAKARA) expression vector containing the *csp*A (cold shock protein A) promoter, the N-terminal fused CDS of the chaperone Trigger Factor (TF) and an N-terminal hexahistidine (6× His) tag. The cloned sequence was confirmed by direct sequencing from MWG (Heidelberg, Germany) and the obtained construct was referred to as p*Cold-TF-gal-8*. The constructed recombinant p*Cold-TF-gal-8* plasmid was used to express *Pl*-GAL-8 fusion protein in *E. coli* BL21 (DE3) (Novagen), after induction with 1 mM isopropyl-b-D-thiogalactopyranoside (IPTG, Sigma) at 15 ^o^C for 48 h. Cells were harvested by centrifugation at 4,000 rpm at 4 ^o^C for 10 min, re-suspended in lysis buffer (50 mM Tris, 0.5 M NaCl, 2 mM PMSF and 5 mM imidazole, pH 7.5), disrupted by sonication and centrifuged at 10,000 g at 4 ^o^C for 30 min. The supernatant was applied to a Ni-nitrilotriacetate column (Invitrogen), pre-equilibrated with lysis buffer. After washing of the column with: 50 mM Tris, 50 mM NaCl, 20 mM imidazole, pH 7.5), 1 ml fractions were eluted in: 50 mM Tris, 100 mM NaCl, 300 mM imidazole, pH 7.5. The fractions containing the recombinant protein (measured by Bradford and 7.5% SDS–PAGE) were pooled and concentrated by centrifugation in Amicon Ultra centrifugal filter units, 50 kDA cut-off (www.millipore.com). After dialysis against 50 mM Tris, 50 mM NaCl, pH 7.5, the protein concentration was determined using a Bradford assay (Bio-Rad) and the purity analysed on a 7.5% SDS polyacrylamide gel.

### Hemagglutination assay

The carbohydrates-binding activity of the recombinant *Pl*-GAL-8 was assayed by measuring its ability to agglutinate human erythrocytes^51^. Briefly, 50 μL of a 4% v/v suspension of PBS-washed fresh human red blood cells (RBCs) were mixed with 50 μL of serial dilutions of the recombinant *Pl*-GAL-8 in PBS, at concentrations ranging from 0.01 μΜ to 1 μM, and disposed in wells of U-shaped 96-wells microtiter plates. The assay was performed in triplicates for each given concentration, plates were incubated in the dark, at room temperature for 40 min, and results were recorded by macro-photography. Negative controls included: i) denatured recombinant protein (3 min at 100 °C); ii) bovine serum albumin (BSA); iii) expressed and purified unfused trigger factor chaperone protein. To test the carbohydrate specificity, the inhibition of hemagglutination promoted by recombinant *Pl*-GAL-8 (0.25 μM) was tested in the presence of different sugars, including lactose, galactose, glucose, sucrose, maltose, mannose (purchased from Sigma), at concentrations ranging from 1 mM to 20 mM. The percentage of agglutination was determined by measuring the intensity using the ImageJ software.Measurements obtained in arbitrary units are expressed as percentage of agglutination activity. Values are presented as the mean of triplicates from three experiments, with +/− SD.

### Cell adhesion assay

Human hepatoma cells (HepG2), grown in complete medium containing 10% (v/v) Fetal Bovine Serum (FBS), were detached from tissue culture plates and re-suspended (3 × 10^5^ cells/ml) in the absence or presence of soluble recombinant human Galectin-8 (h-Gal-8, R&D Systems) or recombinant *P. lividus* Galectin-8 (r-*Pl*-GAL-8). Cells were seeded (100 μl per well) in 96-well tissue culture flat bottom plates and incubated at 37 °C. After 3 hours, cells were carefully washed three times with serum-free RPMI medium (Gibco, life tech.), and three times with phosphate-buffered saline (PBS). Adherent cells were quantified according to Li *et al*.^52^. Taking advantage of the reported autofluorescence induced by glutaraldehyde, cells were incubated with 100 μl of 0.5% glutaraldehyde in PBS for 5 min at room temperature. After three PBS washes, the 96 well plates were scanned using the Odyssey Infrared Imaging System (LI-COR Biosciences, Lincoln, NE, USA) at 169 μm resolution (medium quality, 3.0 mm focus offset, intensity setting of 7) for 700 nm channel. Fluorenscence was quantified using the application software, version 3.0 (LI-COR, Biosciences).

## Additional Information

**How to cite this article**: Karakostis, K., Costa, C. *et al*. Heterologous expression of newly identified galectin-8 from sea urchin embryos produces recombinant protein with lactose binding specificity and anti-adhesive activity. *Sci. Rep*. **5**, 17665; doi: 10.1038/srep17665 (2015).

## Supplementary Material

Supplementary Information
