# Erratum: Heterologous expression of newly identified galectin-8 from sea urchin embryos produces recombinant protein with lactose binding specificity and anti-adhesive activity

**DOI:** 10.1038/srep19169

**Published:** 2016-01-20

**Authors:** Konstantinos Karakostis, Caterina Costa, Francesca Zito, Valeria Matranga

Scientific Reports 5: Article number: 17665; 10.1038/srep17665 published online: 12072015; updated: 01202016.

The original version of this Article contained a typographical error in the spelling of the author Konstantinos Karakostis, which was incorrectly given as Kostantinos Karakostis. This has now been corrected in both the PDF and HTML versions of the Article.

