# Supplementary material for: Heterologous expression of newly identified galectin-8 from sea urchin embryos produces recombinant protein with lactose binding specificity and anti-adhesive activity
Source: Sci Rep. 2015 Dec 7;5:17665. doi: 10.1038/srep17665 (PMC4671058; doi:10.1038/srep17665)
Supplement: Supplementary Information [file srep17665-s1.pdf]

Heterologous expression of newly identified galectin-8 from sea urchin embryos produces recombinant protein with lactose binding specificity and anti-adhesive activity

Konstantinos Karakostis#, C. Costa#, F. Zito and V. Matranga*

Institute of Biomedicine and Molecular Immunology "A. Monroy",

National Research Council, Palermo, Italy

*****Corresponding author: matranga@ibim.cnr.it

[#] Equally contributed to this study

TABLE I - Members of the galectin family in invertebrates

Name	Species	type	MW (kDa)	Cell types & tissues	Function
GALEC2a_SUBDO[1]	*Suberites domuncula* Porifera	tandem-repeat	33,091	extracted from axial filaments of the spicules	spicule growth, biosilica deposition; self-associate in the Ca2+ presence; co-precipitates with Silicatein.
CaL [2]	*Cinachyrella apion* Porifera	eight subunits of 15.5 kDa	124	extracted and purified from total animals	induces cell death; haemagglutinating activity towards human erythrocytes of all ABO groups.
GCG [3-5]	*Geodia cydonium* Porifera	prototype and chimera oligomers	13,15,16<br>30,46, 62	extracellular fluid of the sponge	binding to terminal blood group A-related saccharides on polylactosamine-type glycans.
HOL-30 [6]	*Halichondria okadai* Porifera	forming dimers of 60KDa	30	extract from homogenised organisms	agglutinate rabbit and human erythrocytes.
Nematogalectin A e Nematogalectin B [7]	*Hydra and Clytia* Cnidaria	prototype forming trimer	28<br>120	proximal tubule (B) and distal tubule (A) (Desmonemes, stenoteles and isorhizas)	nematocyst tubules differentiation.
OvGalBP [8]	*Onchocerca volvulus* Nematode *(filaria)*	tandem repeat	32.5	adult female	specifically bind IgE with a role in the pathophysiology of human filarial infection.
Hco-gal-1, Tco-gal-2 [9]<br><br>Hco-GAL-3a<br>Hco-gal-4 [10]	*Hemonchus contortus* Nematode	tandem repeat	31-35	third stage larvae<br><br>all life cycle stages, adult parasites	antigen of the sheep gastrointestinal nematode parasite.
Tci-gal-1 and -2 [11]	*Teladorsagia circumcincta* Nematode	tandem repeat	31 – 33	third stage larvae	antigen of the sheep gastrointestinal nematode parasite .

N32, LEC-1<br>N16, LEC-6 [12-14]<br>*lec-2–5*<br>lec-8–11<br><br>DC2.3a/LEC-12a<br>LEC-8 [15-17]	*Caenorhabditis elegans*<br>Nematode	Tandem repeat<br><br>tandem repeat<br>chimera<br><br>prototype<br>prototype	32<br>16<br><br><br>31–35<br><br>16-27<br>19,9<br><br>-	adult cuticle<br><br><br><br>--------<br><br>--------<br>--------<br>pharyngeal-intestinal valve and intestinal-rectal valve, weakly in intestine.	hypothesised role in the formation of tight and insoluble epidermal layers.<br>recombinant for LEC-2–4 and LEC-8–11.<br><br><br>hemagglutinating activity against rabbit erythrocytes.<br>host defense against bacterial infection by competition.
Pl-Gal-8	*Paracentrotus lividus*<br>Echinoidea	tandem-repeat	35	Embryonic endodermal cells and secondary mesnchyme cells.<br>Larvae sphinters and gut.	hemoagglutination and<br>cell adhesion.
CvGal [18]<br>CgGal [19]	*Crassostrea virginica*<br>Bivalvia	four CRD<br>130kDa<br><br>prototype	70,9<br><br><br>16.5	hemocytes, palps, gills, mantle, midgut, and rectal tissues.<br>hemocytes, , mantle, gills, digestive gland, gonad, adductor muscle, heart, labial palp.	cell-cell interactions;<br>host-pathogen interactions;<br>Immune system;<br>cell adhesion
AiGal1[20]	*Argopectens irradians*<br>Bivalvia	four CRDs	61.5	haemocytes	immune responses against bacterial infection
SgGal-1 [21]	*Solen grandis*<br>Bivalvia	tandem repeat	47.1	hemocytes, gonad, mantle, muscle,<br>gill and hepatopancreas	pattern recognition receptors (PRRs) in pathogen recognition
PoGal1 [22]<br><br>PoGal2<br>PfGal	*Pinctada fucata*<br>Bivalvia	four CRDs<br><br><br>tandem-repeat<br>tandem-repeat	63.4<br><br><br>38.1<br><br>38,09	up-regulated in digestive gland, mantle, haemocyte, gonad and intestine after *Vibrio alginolyticus* infection.	innate immune response.<br><br>constitutive and inducible acute-phase protein involved in the innate immune response .<br><br>immune response against bacterial infection and clearance of bacterial pathogens.
McGal [23]	*Ruditapes philippinarum*<br>Bivalvia	tandem repeat	33.9	heart, mantle, foot, adductor, palp, siphon tissues and hemocytes	up-regulated after challenge with *Perkinsus olseni* or *Vibrio tapetis* binding to their surface .

BgGal [24]	*Biomphalaria glabrata* Gastropoda	tandem-repeat	32,0	embryonic cell line (Bge), circulating haemocytes	pattern recognition receptor for pathogens(*Schistosoma mansoni*).
CiLgals-a CiLgals-b [25,26]	*Ciona intestinalis* Ascidiacea	Tandem repeat	32.9 37.1	pharynx extracts and hemocytes	Infiammatory responses more probable for CiLgals-a
Dmgal [27]	*Drosophila melanogaster* Diptera	tandem repeat	57,6	Embryonic, larval, and adult somatic and visceral musculature, central nervous system.	Innate immune system.
PpGalec [28]	*Phlebotomus papatasi* Diptera	tandem repeat		larval and pupal stages of development - midgut	interaction with Leishmania protozoan parasites and parasites adherence facilitation to the midgut epithelial cells
OmGalec [29]	*Ornithodoros moubata,* Arachnida	tandem repeat	37,4	all stages of the life cycle adult hemocytes, midguts, and reproductive organs	immunity, interaction with pathogens, and development

Supplementary References

1 Schroder, H. C. *et al.* Co-expression and functional interaction of silicatein with galectin: matrix-guided formation of siliceous spicules in the marine demosponge Suberites domuncula. *The Journal of biological chemistry* **281**, 12001-12009, doi:10.1074/jbc.M512677200 (2006).

2 Medeiros, D. S. *et al.* A lactose specific lectin from the sponge Cinachyrella apion: purification, characterization, N-terminal sequences alignment and agglutinating activity on Leishmania promastigotes. *Comparative biochemistry and physiology. Part B, Biochemistry & molecular biology* **155**, 211-216, doi:10.1016/j.cbpb.2009.10.016 (2010).

3 Pfeifer, K. *et al.* S-type lectins occur also in invertebrates: high conservation of the carbohydrate recognition domain in the lectin genes from the marine sponge Geodia cydonium. *Glycobiology* **3**, 179-184 (1993).

4 Wagner-Hulsmann, C. *et al.* A galectin links the aggregation factor to cells in the sponge (Geodia cydonium) system. *Glycobiology* **6**, 785-793 (1996).

5 Stalz, H. *et al.* The Geodia cydonium galectin exhibits prototype and chimera-type characteristics and a unique sequence polymorphism within its carbohydrate recognition domain. *Glycobiology* **16**, 402-414, doi:10.1093/glycob/cwj086 (2006).

6 Kawsar, S. M. *et al.* Isolation, purification, characterization and glycan-binding profile of a d-galactoside specific lectin from the marine sponge, Halichondria okadai. *Comparative biochemistry and physiology. Part B, Biochemistry & molecular biology* **150**, 349-357, doi:10.1016/j.cbpb.2008.04.004 (2008).

7 Hwang, J. S. *et al.* Nematogalectin, a nematocyst protein with GlyXY and galectin domains, demonstrates nematocyte-specific alternative splicing in Hydra. *Proceedings of the National Academy of Sciences of the United States of America* **107**, 18539-18544, doi:10.1073/pnas.1003256107 (2010).

8 Klion, A. D. & Donelson, J. E. OvGalBP, a filarial antigen with homology to vertebrate galactoside-binding proteins. *Molecular and biochemical parasitology* **65**, 305-315 (1994).

9 Greenhalgh, C. J., Beckham, S. A. & Newton, S. E. Galectins from sheep gastrointestinal nematode parasites are highly conserved. *Molecular and biochemical parasitology* **98**, 285-289 (1999).
10 Greenhalgh, C. J., Loukas, A., Donald, D., Nikolaou, S. & Newton, S. E. A family of galectins from Haemonchus contortus. *Molecular and biochemical parasitology* **107**, 117-121 (2000).
11 Newton, S. E., Monti, J. R., Greenhalgh, C. J., Ashman, K. & Meeusen, E. N. cDNA cloning of galectins from third stage larvae of the parasitic nematode Teladorsagia circumcincta. *Molecular and biochemical parasitology* **86**, 143-153 (1997).
12 Ahmed, H. *et al.* Novel carbohydrate specificity of the 16-kDa galectin from Caenorhabditis elegans: binding to blood group precursor oligosaccharides (type 1, type 2, Talpha, and Tbeta) and gangliosides. *Glycobiology* **12**, 451-461 (2002).
13 Hirabayashi, J., Satoh, M., Ohyama, Y. & Kasai, K. Purification and characterization of beta-galactoside-binding proteins from Caenorhabditis elegans. *Journal of biochemistry* **111**, 553-555 (1992).
14 Hirabayashi, J., Ubukata, T. & Kasai, K. Purification and molecular characterization of a novel 16-kDa galectin from the nematode Caenorhabditis elegans. *The Journal of biological chemistry* **271**, 2497-2505 (1996).
15 Nemoto-Sasaki, Y. *et al.* Caenorhabditis elegans galectins LEC-1-LEC-11: structural features and sugar-binding properties. *Biochimica et biophysica acta* **1780**, 1131-1142, doi:10.1016/j.bbagen.2008.07.003 (2008).
16 Nemoto-Sasaki, Y. *et al.* The DC2.3 gene in Caenorhabditis elegans encodes a galectin that recognizes the galactosebeta1-->4fucose disaccharide unit. *Biological & pharmaceutical bulletin* **34**, 1635-1639 (2011).
17 Ideo, H. *et al.* A Caenorhabditis elegans glycolipid-binding galectin functions in host defense against bacterial infection. *The Journal of biological chemistry* **284**, 26493-26501, doi:10.1074/jbc.M109.038257 (2009).
18 Tasumi, S. & Vasta, G. R. A galectin of unique domain organization from hemocytes of the Eastern oyster (Crassostrea virginica) is a receptor for the protistan parasite Perkinsus marinus. *Journal of immunology* **179**, 3086-3098 (2007).
19 Yamaura, K., Takahashi, K. G. & Suzuki, T. Identification and tissue expression analysis of C-type lectin and galectin in the Pacific oyster, Crassostrea gigas. *Comparative biochemistry and physiology. Part B, Biochemistry & molecular biology* **149**, 168-175, doi:10.1016/j.cbpb.2007.09.004 (2008).
20 Song, B. *et al.* [Isoflurance-based intravenous and inhalation combined anesthesia versus low-dose-ketamine-based total intravenous anesthesia for valvuloplasty in minipigs]. *Sichuan da xue xue bao. Yi xue ban = Journal of Sichuan University. Medical science edition* **41**, 858-861 (2010).
21 Yang, J. *et al.* A sigma-class glutathione S-transferase from Solen grandis that responded to microorganism glycan and organic contaminants. *Fish & shellfish immunology* **32**, 1198-1204, doi:10.1016/j.fsi.2012.03.010 (2012).
22 Zhang, D. *et al.* A multidomain galectin involved in innate immune response of pearl oyster Pinctada fucata. *Developmental and comparative immunology* **35**, 1-6, doi:10.1016/j.dci.2010.08.007 (2011).
23 Kim, J. Y., Kim, Y. M., Cho, S. K., Choi, K. S. & Cho, M. Noble tandem-repeat galectin of Manila clam Ruditapes philippinarum is induced upon infection with the protozoan parasite Perkinsus olseni. *Developmental and comparative immunology* **32**, 1131-1141, doi:10.1016/j.dci.2008.03.002 (2008).
24 Yoshino, T. P., Dinguirard, N., Kunert, J. & Hokke, C. H. Molecular and functional characterization of a tandem-repeat galectin from the freshwater snail Biomphalaria

glabrata, intermediate host of the human blood fluke Schistosoma mansoni. *Gene* **411**, 46-58, doi:10.1016/j.gene.2008.01.003 (2008).

25 Satou, Y. *et al.* A cDNA resource from the basal chordate Ciona intestinalis. *Genesis* **33**, 153-154, doi:10.1002/gene.10119 (2002).

26 Vizzini, A. *et al.* Inducible galectins are expressed in the inflamed pharynx of the ascidian Ciona intestinalis. *Fish & shellfish immunology* **32**, 101-109, doi:10.1016/j.fsi.2011.10.028 (2012).

27 Pace, K. E. *et al.* Characterization of a novel Drosophila melanogaster galectin. Expression in developing immune, neural, and muscle tissues. *The Journal of biological chemistry* **277**, 13091-13098, doi:10.1074/jbc.M112105200 (2002).

28 Kamhawi, S. *et al.* A role for insect galectins in parasite survival. *Cell* **119**, 329-341 (2004).

29 Huang, X. *et al.* Molecular characterization and oligosaccharide-binding properties of a galectin from the argasid tick Ornithodoros moubata. *Glycobiology* **17**, 313-323, doi:10.1093/glycob/cwl070 (2007).
